# Laser-Based Mid-Infrared Spectroscopy for Monitoring Temperature-Induced Denaturation of Bovine Serum Albumin and De-/Stabilization Effects of Sugars

**DOI:** 10.1021/acs.analchem.3c00489

**Published:** 2023-04-03

**Authors:** Shilpa Vijayakumar, Jeremy Rowlette, Andreas Schwaighofer, Bernhard Lendl

**Affiliations:** † Research Division of Environmental Analytics, Process Analytics and Sensors, Institute of Chemical Technologies and Analytics, TU Wien, Getreidemarkt 9, Vienna 1060, Austria; ‡ DRS Daylight Solutions Inc., San Diego, California 92127, United States

## Abstract

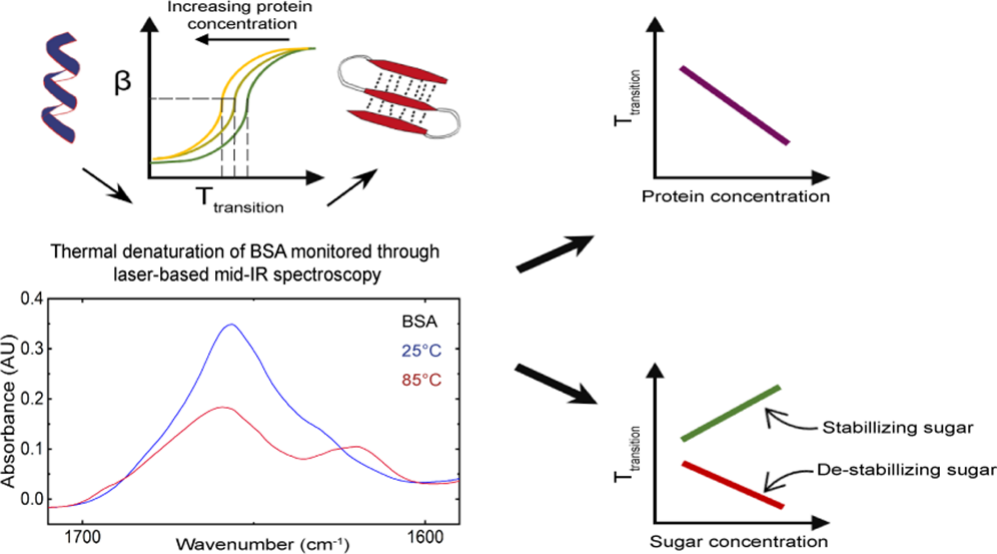

Stability of high-concentration protein formulations is considered a major challenge in current biopharmaceutical development. In this work, we introduce laser-based mid-infrared (IR) spectroscopy as a versatile technique to study the effect of protein concentration and presence of sugars on the thermal denaturation of the model protein bovine serum albumin (BSA). Many analytical techniques struggle to characterize the complex structural transition that occurs during protein denaturation. To this end, a commercially available laser-based mid-IR spectrometer equipped with a customized flow cell was employed to record IR spectra of BSA in the temperature range of 25–85 °C. The temperature perturbation induces a conformational change from a native α-helical to an intermolecular β-sheet secondary structure in BSA. Systematic investigation of the concentration dependence of the α–β transition temperature between 30 and 90 mg mL^–1^ shows a trend of decreasing denaturation temperatures at higher BSA concentrations. In-depth chemometric analysis by a multivariate curve resolution-alternating least squares (MCR-ALS) analysis of the spectra, suggested the formation of not one but two intermediates in the denaturation of BSA. Subsequently, the impact of sugars on denaturation temperatures was investigated, revealing both stabilizing (trehalose, sucrose, and mannose) and destabilizing (sucralose) effects, illustrating the applicability of this method as an investigative tool for stabilizers. These results highlight the potential and versatility of laser-based IR spectroscopy for analysis of protein stability at high concentrations and varying conditions.

## 1 Introduction

The pharmaceutical and food industries often use high concentrations of proteins in their formulations.^1^ However, at high concentrations, proteins suffer from increased viscosity and a tendency to aggregate.^1,2^ Moreover, upon exposure to external perturbations such as temperature changes, pH variations, or chemical agents, many proteins are prone to unfolding,^3,4^ which increases their propensity to aggregate.^3,5^ In this context, it has been shown that unfolding, aggregation, and structural change in proteins, collectively described by the term denaturation, lead to the loss of function in proteins.^4,6,7^ Hence arises the need for stabilizers in the course of formulation and storage of proteins,^1,5,8^ along with suitable analytical methods for characterization.

Saccharides are commonly used as stabilizing agents to address the problem of protein denaturation.^9^ Trehalose, for example, is a disaccharide also found in nature in plants and animals that has been known to protect cells against drying and high temperatures. Other frequently used stabilizing carbohydrates include sucrose, glucose, and inulin among others.^9,10^ However, it has also been reported that there are structure-similar compounds, such as the artificial sweetener sucralose, that show an opposite effect and result in thermal destabilizing effects on proteins.^11^ Choosing a suitable stabilizer for a given purpose hence requires careful consideration of the mechanisms of interaction of sugars with proteins. Many theories have been propounded to explain the stabilizing action of carbohydrates including water entrapment (preferential exclusion), water replacement, sugar glass formation, and combinations of these effects; however, a comprehensive understanding of the effect on a molecular level is still unclear.^12−16^ For investigation of these effects, bovine serum albumin (BSA) is a favored model protein, which has been used for diverse biophysical, biochemical, and physiochemical studies including investigations of protein stabilization by diverse carbohydrates.^5,13^ Numerous analytical methods have been used to research the issue, ranging from depolarized light scattering spectroscopy (DLS) and infrared spectroscopy (IR) to differential scanning calorimetry (DSC) and cold-spray ionization time-of-flight mass spectrometry (CSI TOF-MS).^13,15−18^

Of the several analytical tools available to study protein structure and denaturation, mid-IR spectroscopy provides benefits in terms of flexible sample preparation, the possibility to analyze turbid liquids, and applicability in a wide range of protein concentration.^19−21^ This spectroscopic technique detects the molecular vibrations of molecules in a label-free and nondestructive way. For protein analysis, the most significant spectral feature in the mid-IR range is the amide I band between 1600 and 1700 cm^–1^, which originates from the C=O stretching and N–H in-phase bending vibration of the amide group. It is a sensitive reporter of the secondary structure of proteins, as differing patterns of hydrogen bonding, dipole–dipole interactions, and geometric orientations in α helices, β-sheets, turns, and random coil structures lead to characteristic band shapes and positions.^20,22^

Mid-IR spectroscopy is particularly sensitive to β-sheet structures that are associated with protein denaturation.^23^ The most common implementation of IR spectroscopy, Fourier transform (FT)-IR spectroscopy suffers from the low emission intensity of the employed thermal light source (globar). Due to an intense absorption of the HOH bending vibration of water overlapping with the amide I band, the transmission path length is limited to ∼10 μm for protein analysis in aqueous solution in order to avoid total absorption,^24,25^ resulting in impaired sample handling particularly of high-viscosity samples such as protein–sugar mixtures. To overcome this limitation, in recent years, mid-IR transmission setups based on quantum cascade lasers (QCLs) were developed for protein analysis and were successfully employed for monitoring protein structural changes induced by external perturbations.^26−28^ Due to their spectral power densities that are higher by a factor of 10^4^ compared to thermal light sources, the optical path for transmission measurements could be increased by a factor of 4–5 compared with conventional FTIR spectroscopy. These improvements allowed robust and sensitive spectral acquisition of even highly concentrated protein solutions with increased viscosity. In recent years, laser-based IR spectroscopy has been successfully employed to study conformational changes of proteins induced by different means of external perturbation.^25,29−31^

In this work, a commercial laser-based mid-IR spectrometer equipped with a custom-made temperature-stabilized transmission flow cell was employed to probe the concentration-dependent thermal denaturation of BSA. Furthermore, the stabilizing effect of four sugars, trehalose, sucrose, mannose, and sucralose, on the thermal stability of BSA was systematically investigated.

## 2 Materials and Methods

### 2.1 Preparation of Protein Solutions

BSA, sucrose, and sucralose were procured from Sigma-Aldrich (St. Louis, MO). Mannose and trehalose were purchased from Merck & Co. Inc. (Kenilworth, NJ). BSA concentrations of ∼30, 40, 60, 80, and 90 mg mL^–1^ were prepared by dissolving appropriate amounts of lyophilized BSA powder in milliQ water. For preparation of protein–sugar solutions, 0.1, 0.4, and 0.7 M solutions of trehalose, sucrose, and mannose were prepared, to which BSA was added to obtain constant protein concentrations of 40 mg mL^–1^. For sucralose, the highest concentration of sugar used was limited to 0.5 M due to its low solubility in water. The protein–sugar solutions were hence prepared with sucralose concentrations of 0.1, 0.3, and 0.5 M. Protein concentrations were spectrophotometrically verified by UV spectroscopy at 280 nm using a Cary 50 Bio UV–vis spectrometer (Agilent Technologies, Santa Clara, CA). UV spectra of the prepared protein and protein–sugar solutions were recorded using a 1 mm cuvette and an absorption coefficient of 42 925 M^–1^ cm^–1^.^32^

### 2.2 Laser-Based Infrared Spectroscopy

The commercially available ChemDetect Analyzer (DRS Daylight Solutions, San Diego) was used for recording laser-based IR spectra in the spectral range of 1350–1750 cm^–1^. An external water-cooling unit was operated at 17 °C in order to ensure thermal stabilization of the laser head during operation. Spectra were acquired with the ChemDetect software package. Samples were injected into a custom-made temperature-controlled transmission flow cell with a path length of 25 μm. Temperature control of the cell was achieved using a Meerstetter Engineering thermoelectric cooler (TEC) controller (TEC 1091).

For monitoring thermally induced protein denaturation, triplicate measurements were performed at every protein concentration. After injection of the protein solution at room temperature into the measurement cell, the temperature was increased at a rate of 0.05 °C/s from 25 to 85 °C. 40 IR spectra were collected in every measurement run, each obtained by averaging 60 scans (30 s). For referencing, temperature-induced changes in the IR spectrum of water were recorded with the same temperature ramp. For the protein–sugar tertiary solutions, duplicate measurements were performed at each sugar concentration. Also, here, temperature-dependent reference spectra were recorded from the corresponding plain sugar solution. To study the influence of sugar concentration on the water background, sugar spectra at varying concentrations were collected at 25 °C.

### 2.3 IR Spectra Processing

The spectra were processed using MATLAB R2021b. For obtaining the IR absorbance spectra depicting the temperature-induced secondary structure change of proteins, the recorded water spectrum at each temperature was then subtracted from the protein spectrum at the matching temperature to remove the contribution of spectral changes effected in water with varying temperature. This was achieved by an iterative algorithm that optimized the subtraction factor to obtain an equal slope for all spectra of an experiment between 1715 and 1750 cm^–1^.^34^ Similarly, temperature-dependent sugar spectra were subtracted from the protein–sugar spectra. For subsequent evaluation, second-derivative spectra were calculated along with a second-order Savitzky–Golay filter of window size 15 for noise reduction.^35^ Although the collected spectra covered both the amide I and amide II bands as seen in Figure S1, only the amide I band was used for further processing. A schematic of the performed processing steps is shown in Figure S2.

### 2.4 MCR-ALS

Multivariate curve resolution (MCR) is a soft-modeling technique that can be used to retrieve pure component profiles from a diverse set of scientific data, including pure spectra from spectroscopic data.^36^ It is applicable without prior knowledge of the system and is therefore suitable to study the presence and development of intermediates whose concentration profiles and spectra are unknown.^30,37,38^ MCR applied to spectroscopy takes the form
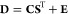
1which can be regarded as an extension of the Beer–Lambert law.^39^ Matrix **D** is constituted of row-wise spectra and is decomposed into a concentration matrix, **C**, and a spectral matrix, **S**^T^, along with the residual matrix **E**. The alternate least-squares (ALS) algorithm iteratively solves for **C** and **S** until the standard deviation of residuals in consecutive iterations falls below a certain threshold, which is the convergence criteria. Through the enforcement of soft and hard constraints, rotational ambiguity in the solutions is reduced. The lack of fit (LOF) is calculated by
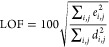
2wherein *d*_*i,j*_ are elements of the data matrix **D** and *e*_*i,j*_ are the corresponding residuals. LOF can be used as a measure of the quality of the fit determined by the ALS algorithm along with the percentage of variance explained.

To obtain better temperature resolution for MCR-ALS, a protein denaturation data set at a concentration of 60 mg mL^–1^ was recorded at a slower heating rate of 0.01 °C/s. MCR-ALS was performed with an open-source toolbox that solves the MCR model through the ALS algorithm and can be used with MATLAB.^40^ The toolbox allows users to select the number of components either manually or using singular value decomposition (SVD), to make initial estimates for the selected number of components and to apply hard constraints to the model. MCR-ALS was performed to investigate the presence of intermediates as suggested in previous studies.^5,41^ The number of components was varied between two (no intermediates) and five (three intermediates). For assessing the optimum number of components, the shape and intensity of the error matrix **E**, the LOF, and the percentage of explained variance were evaluated. Initial estimates of the individual components were obtained using the graphical interface for evolving factor analysis (EFA) provided in the MCR-ALS GUI, with an eigen value cutoff of 1.^42^ Finally, a non-negativity constraint was applied in the spectral and concentration modes, a unimodality constraint was applied on the native α-helical and β-sheet structure, and a closure constraint was applied to ensure the mass balance of all of the species.^37^

## 3 Results and Discussion

### 3.1 Temperature-Induced Denaturation of BSA

**Figure 1:**
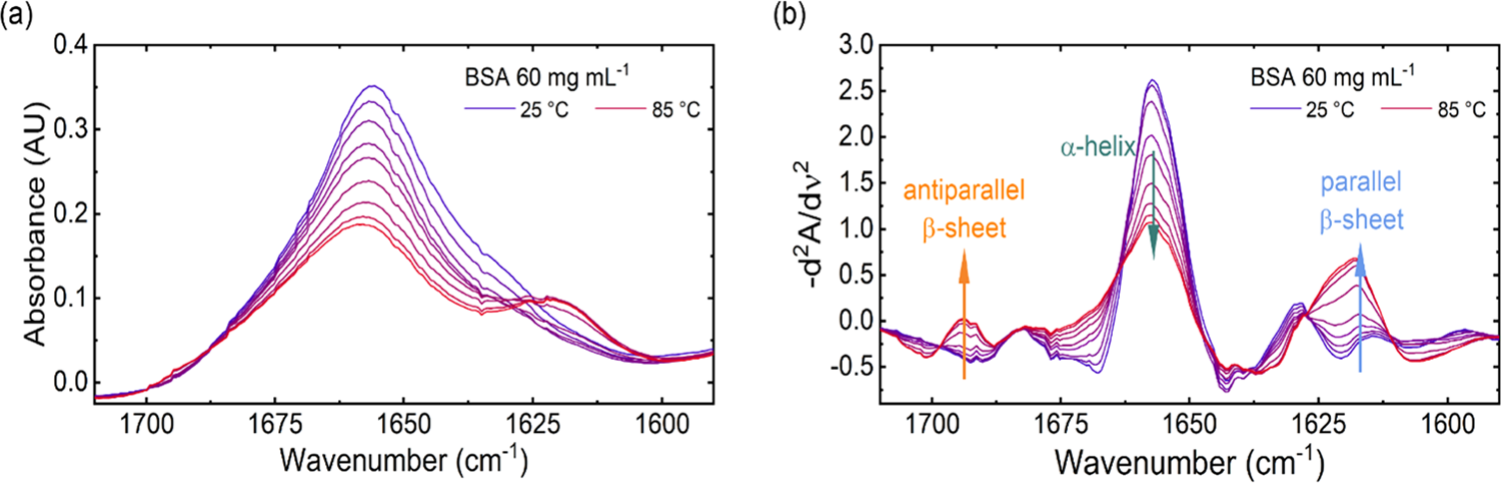
(a) IR absorbance spectra and (b) inverted second-derivative spectra of BSA at a concentration of 60 mg mL^–1^. Panels (a) and (b) show the formation of intermolecular β-sheets and the depletion of the α-helical structure of BSA due to temperature-induced aggregation.

Laser-based IR spectra of the temperature-induced change of BSA were recorded between 25 and 85 °C. In Figure 1a, the QCL-IR absorption spectra of the amide I region of a 60 mg mL^–1^ BSA solution are exemplarily shown. The QCL-IR spectrum at 25 °C shows a band maximum at 1655 cm^–1^, characteristic of the IR signature of BSA that features a predominantly α-helical secondary structure.^43^ On gradually increasing the temperature of the protein solutions, a reduction in the band height at 1655 cm^–1^ was observed, along with the appearance of an absorption band at 1617 cm^–1^ and a smaller shoulder at 1692 cm^–1^. This emerging band shape is assigned to the formation of parallel and antiparallel intermolecular β-sheets that are typical for protein aggregation processes.^20^ The spectral changes were subsequently evaluated using second-derivative spectra (Figure 1b) to make them more apparent.

### 3.2 Dependence of Denaturation on Protein Concentration

**Figure 2:**
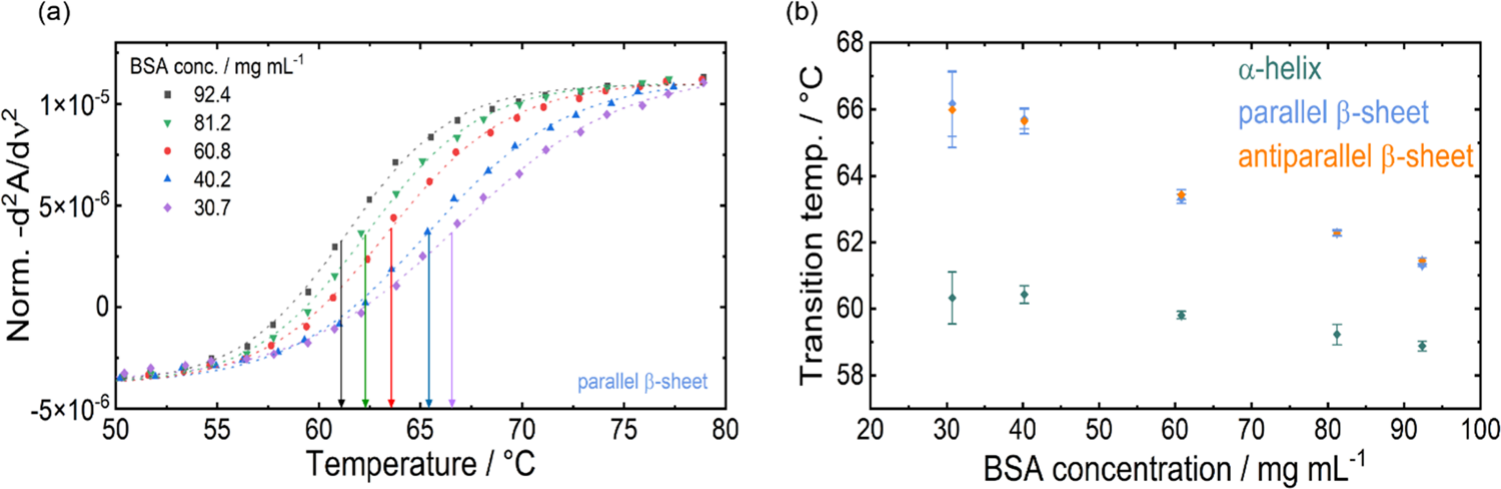
(a) Normalized second-derivative band heights at 1617 cm^–1^ attributed to parallel β-sheets plotted against temperature for BSA concentrations ranging from 30 to 90 mg mL^–1^. The transition temperatures of sigmoidal fits (dashed lines) are indicated by arrows. (b) Transition temperatures of individual secondary structure elements plotted for different concentrations.

The progression of temperature-induced denaturation of BSA was investigated at protein concentrations of 30, 40, 60, 80, and 90 mg mL^–1^. For this purpose, band heights of the α-helical as well as parallel and antiparallel β-sheet secondary structure elements were evaluated in the second-derivative spectra. Figure 2a shows the concentration-normalized second-derivative band heights of the parallel β-sheets plotted against temperature for each protein concentration. The data points were fitted with a Boltzmann function for sigmoidal line shapes and the obtained points of inflection were deemed as denaturation temperatures. Average transition temperatures and statistical data of triplicate measurements are shown in Figure 2b and collected in Table S1. Here, it can be observed that the denaturation temperature decreases with increasing protein concentrations, within the investigated concentration range. Because of this increase of the denaturation temperature at lower temperatures, there followed a limit for the lowest accessible BSA concentration whose denaturation temperature could be investigated using the employed custom-made flow-through transmission cell. Thus, an upper limit of 85 °C was imposed due to the proximity to the boiling point of water at 100 °C.

This effect is also reflected by the increasing values for the standard deviation of the denaturation temperature at lower BSA concentrations (Figure 2b). On the high-concentration end, denaturation measurements above 92.4 mg mL^–1^ could not be reproducibly performed because gelation of the protein occurred within the investigated temperature range,^44^ which lead to leakage of the cell.

The found tendency of an earlier onset of protein denaturation at increased concentrations was mentioned in other works,^21,45^ but was here investigated systematically for the first time by IR spectroscopy. This behavior suggests a link between native reversible self-association of protein molecules due to macromolecular crowding with irreversible unfolding (i.e., denaturation) and that these effects affect each other, which is in accordance with earlier findings.^1,21,46^

IR spectroscopy enables to simultaneously monitor the progression of multiple secondary structure elements. Consequently, it was possible to independently follow the decrease in α-helical content and formation of parallel and antiparallel β-sheet structures. Figure 2b shows the evaluated transition temperatures of the denaturation measurements at different BSA concentrations. As expected, the parallel and antiparallel β-sheet structures show the same transition temperatures. In the investigated concentration range, the transition temperature decreases from 66 °C at 30.7 mg mL^–1^ to 61.5 °C at 92.7 mg mL^–1^. The transition temperatures of the α-helix were found to be at lower temperatures and are decreasing at a lower slope. Here, the values decrease from 60.3 °C at 30.7 mg mL^–1^ to 58.8 °C at 92.7 mg mL^–1^. These transition temperatures for α-helix correspond well to the temperature of 60 °C that is generally reported as the onset of irreversible denaturation of BSA.^3,41,47^

As shown in Figure 2b, the change in transition temperature with BSA concentration is more pronounced for β-sheets than for α-helices. While the decrease of transition temperature between lowest and highest concentration is 4.5 °C for β-sheets, it only amounts to 1.5 °C for α-helices. This behavior indicates that the concentration has a more pronounced influence on the formation of intermolecular β-sheets than on the unfolding of α-helices. A comparable distinct influence of the concentration on formation of intermolecular β-sheet structures has been described for poly-l-lysine (PLL).^29,48^

**Figure 3:**
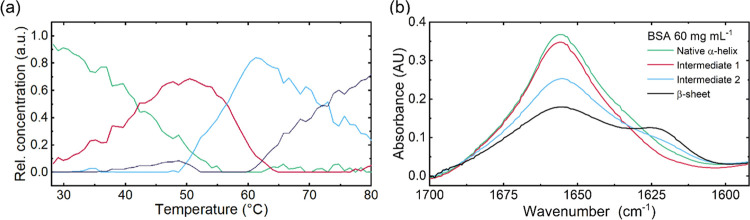
(a) Concentration profiles of the four secondary structure components resolved through MCR-ALS versus temperature. (b) Spectral profiles of the four components in the thermal denaturation of BSA.

An interesting finding is the difference of the transition temperatures of α-helices and β-sheets, which is present in the entire concentration range. This gap suggests that the disintegration of α-helices precedes the formation of β-sheets and that this transformation does not occur directly, but there exist one or more intermediate structures. Given this gap, the existence of a transitional stage would also account for an even mass balance during denaturation from an α-helical to a β-sheet structure. Such intermediate structures in thermal denaturation of BSA have been previously proposed^5,35,49^ but could not be directly resolved in the present work by examining second-derivative spectra. MCR-ALS was hence used to validate the emergence of one or more intermediate structures. To determine the number of intermediates, MCR-ALS was first performed on a two-component system (no intermediates) and components were gradually increased up to five (three intermediates) while comparing the LOF and percentage of variance explained in each case. The determination of the number of components in an MCR model is delicate. While too few components can lead to the loss of information and an incomplete model, including too many components results in overfitting.^50^ Hence a careful consideration of the figures of merit in the case of the different component models was made. The error matrices obtained for the individual models can be seen in Figure S3. It is evident that the error in a four-component system is rather low (±3 mAU) and appears as random noise, whereas in the two- and three-component systems, the residuals in the error matrix are higher and appear to be more structured. Although increasing the number of components up to four improved the LOF and percentage of explained variance, on further increasing the number of components from four to five, these figures of merit started to deteriorate (shown in Figure S4e,i). An overlay of the concentration and spectral profiles of the components obtained for three- and four-component models can be seen in Figure S5. While the absorbance spectra appear to be fairly similar, the differences between the intermediates in wavenumber regions 1625–1647 and 1664–1698 cm^–1^ are visible in the second-derivative spectra as shown in Figure S6. Domínguez-Vidal^41^ modeled the denaturation of BSA at 60 °C using three components in MCR, reasoning that the two-dimensional correlation spectroscopy data of the residuals of the two-component system presented was not devoid of information. However, the temperature was limited to 60 °C in those experiments, contrary to the extended range up to 85 °C presented here. On observing the concentration profiles of the various components in the four-component model detailed in this work (Figure 3a), the second intermediate can be seen to be replaced by the well-resolved β-sheet component only beyond 60 °C, further supporting the possibility of the presence of a second intermediate in the temperature range of the performed experiments. While augmenting the data matrix with additional information from other spectroscopic techniques could help to further resolve the highly complex temperature region between 40 and 65 °C, it is out of scope of this paper. Within this evaluation, the optimal number of resolved components based on the various figures of merit and structure of the error matrix, was hence determined to be four for modeling the thermal denaturation of BSA. The resulting spectral and concentration profiles are presented in Figure 3.

Chemometric analysis of the four-component model yielded two components with spectral profiles resembling an α-helical (green and red) structure and two showing β-sheet (blue and black) elements (Figure 3b). The green and black concentration profiles (Figure 3a) respectively match the expected behavior of the initial α-helical and final parallel β-sheet structural elements during denaturation.^41^ The native α-helical structure component (green) is replaced by the first intermediate component (red) at low temperatures (<50 °C), which is then converted into the second intermediate (blue) and lastly the final β-sheet structure component at temperatures above 60 °C.

### 3.3 Effect of Sugars on Protein Denaturation Temperature

The effect of varying concentrations of different sugars on protein denaturation was investigated. In this regard, denaturation measurements were performed at BSA concentrations of 40 mg mL^–1^ in the presence of trehalose, sucrose, and mannose at concentrations of 0.1, 0.4, and 0.7 mol L^–1^ and sucralose at concentrations of 0.1, 0.3, and 0.5 mol L^–1^. The two disaccharides, trehalose and sucrose, are well-studied additives that have shown to increase the thermostability of proteins.^13,47,51,52^ For the monosaccharide mannose, an increase of stability has been suggested by molecular dynamics simulations.^53^ The destabilizing effect of sucralose was observed by circular dichroism (CD) spectroscopy of BSA and time-resolved fluorescence spectroscopy.^11^

**Figure 4:**
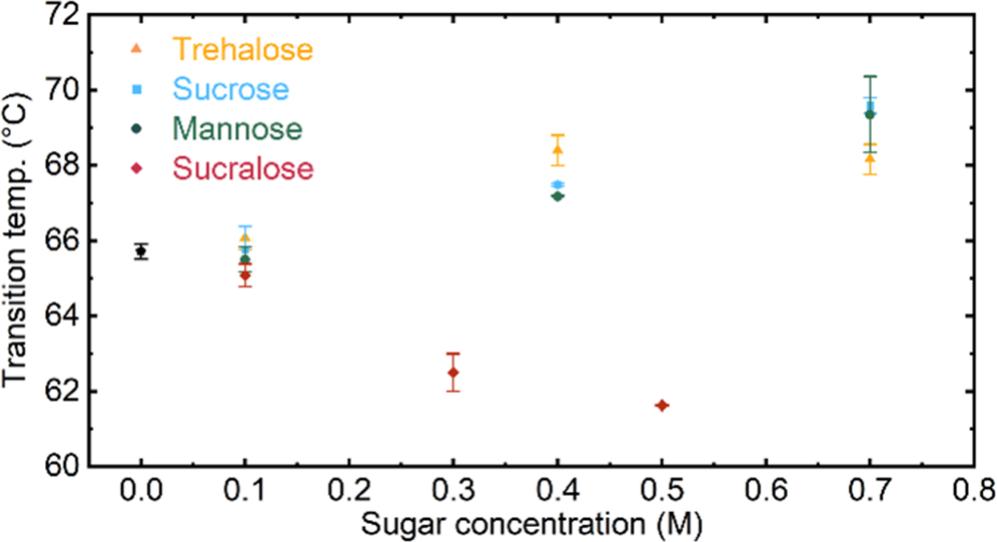
Dependence of thermal denaturation temperatures of BSA on the concentration of sugar type and concentration present in the solution.

The IR spectra of trehalose, sucrose, mannose, and sucralose at varying concentrations are depicted in Figure S7. Differences in their spectra are accounted for by taking a new reference measurement before the start of each experiment with the protein–sugar solutions. In the IR spectra of the denaturation measurements of the BSA–sugar solutions, the presence of sugars in any concentration did not affect the band positions in the spectra of BSA, as exemplarily depicted in Figure S8. However, there could be observed a change of progression of the unfolding of BSA while increasing temperature. In ensuing investigations of the denaturation progression, the second-derivative band height of the band at 1617 cm^–1^ was evaluated and fitted with a sigmoidal band shape to obtain the transition temperature of the parallel β-sheets.^29^ As shown in Figure 4, there can be observed a change of the transition points in the presence of the sugars. In case of trehalose, sucrose, and mannose, higher denaturation temperatures are obtained compared to pure BSA solutions, thus confirming the stabilizing effect of these sugars. Protein–sucralose solutions however exhibit a reduced denaturation temperature in comparison to pure BSA solutions, suggesting the destabilizing action of sucralose.

Furthermore, it can be observed that an increase of the sugar concentrations leads to advancing of the stabilizing effect (or destabilizing effect for sucralose) on BSA as reported before.^13,47^ In case of trehalose, maximum stabilization appears to be reached already at a concentration of 0.4 M, whereas for sucrose and mannose the transition temperatures further increase at 0.7 M. Various studies reported higher effectiveness at lower concentrations of trehalose in thermal stabilization of proteins compared to sucrose. Following the preferential exclusion hypothesis, this discrepancy has been attributed to the differential ability to form intramolecular hydrogen bonds. Trehalose forms fewer internal hydrogen bonds and therefore is available for stronger interaction with water molecules^54^ which preferentially excludes it from the protein hydration shell.^15,55,56^ Also, for sucralose, an increase of the destabilization effect could be observed at higher concentrations. The rather contradictory behavior of sucralose to its naturally occurring disaccharide counterpart, sucrose, has been ascribed to strong electrostatic interactions due to its highly polar nature. Halogenation further alters the hydrogen-bonding capacity of the molecule and enhances the hydrophobicity of sucralose.^11,57^

## 4 Conclusions

Thermal denaturation of BSA was investigated at different protein concentrations and in the presence of different sugars using laser-based IR spectroscopy. This technique provided detailed insight into the concentration dependence of protein denaturation, with a unique emphasis on higher protein concentrations and temperatures. Upon gradual increase of the temperature between 25 and 85 °C, the spectra revealed a decrease of α-helix and increase of parallel and antiparallel β*-*sheet secondary structures and decreasing transition temperatures with increasing BSA concentrations. Chemometric analysis by MCR-ALS exposed the existence of intermediate structures in the denaturation of BSA. In this regard, IR spectroscopy has proven to be a valuable tool for analysis of proteins at high concentration, which is a constraint for other analytical techniques such as DSC and CD spectroscopy.^19,21^ Performed studies focusing on the effect of sugars on the stability of protein solutions determined that trehalose, sucrose, and mannose provide stabilization to the protein while sucralose has a contrasting destabilizing effect.

These findings show that laser-based mid-IR spectroscopy poses as a practical and versatile tool to study the stability of protein formulations and effectiveness of different stabilizers in pharmaceutical and food industry. The analysis potential is not limited to temperature-induced denaturation and can be extended to different denaturing agents including pH changes, salts, and chemical denaturants.

## Supporting Information Available

Transition temperatures for the various protein concentrations used (Table S1); spectra of BSA in the full wavenumber range recorded and of the various sugars used (Figures S1, S7, and S8); graphical representation of the processing steps used (Figure S2); and additional information about the decision process for the number of components in the MCR-ALS model (Figures S3–S6) (PDF)
