# Supplementary material for: Laser-Based Mid-Infrared Spectroscopy for Monitoring Temperature-Induced Denaturation of Bovine Serum Albumin and De-/Stabilization Effects of Sugars
Source: Anal Chem. 2023 Apr 3;95(15):6441–7. doi: 10.1021/acs.analchem.3c00489 (PMC10116488; doi:10.1021/acs.analchem.3c00489)
Supplement: Supplementary file 1 — ac3c00489_si_001.pdf [file ac3c00489_si_001.pdf]

Supporting Information

Laser-Based Mid-IR Spectroscopy for Monitoring Temperature-Induced Denaturation of BSA and De-/Stabilization Effects of Sugars

Shilpa Vijayakumar[1], Jeremy Rowlette[2], Andreas Schwaighofer[1,*] and Bernhard Lendl[1,*]

[1] Research Group Process Analytics, Institute of Chemical Technologies and Analytics, Technische Universität Wien, Getreidemarkt 9, Vienna, 1060, Austria.

[2] DRS Daylight Solutions Inc., San Diego, CA 92127, USA.

Table of Content

Table S1: Transition temperature for different bands of interest in the denaturation spectra of BSA

Figure S1: Full range spectra of BSA measured by ChemDetect

Figure S2: Processing steps for IR spectra

Figure S3: 3D error matrix after MCR-ALS

Figure S4: Variation of LOF and percentage explained variance with number of components

Figure S5: Comparison of the spectral and concentration profiles of 3 and 4 component MCR models

Figure S6: Comparison of the second derivative spectra of 3 and 4 component MCR models

Figure S7: Concentration dependent spectra of sugars

Figure S8: Temperature dependent spectra of sugars

Table S1: Transition temperatures for different bands of interest in the denaturation spectra of BSA

BSA concentration (mg mL^{-1})	T_α (°C)	$T\beta$ *parallel* (°C)	$T\beta$ *antiparallel* (°C)
30.7	60.3±0.7	66.1±0.9	65.9±0.9
40.2	60.4±0.2	65.7±0.2	65.6±0.3
60.8	59.8±0.1	63.3±0.1	63.4±0.1
81.2	59.2±0.3	62.2±0.1	62.2±0.1
92.3	58.8±0.1	61.3±0.1	61.4±0.1

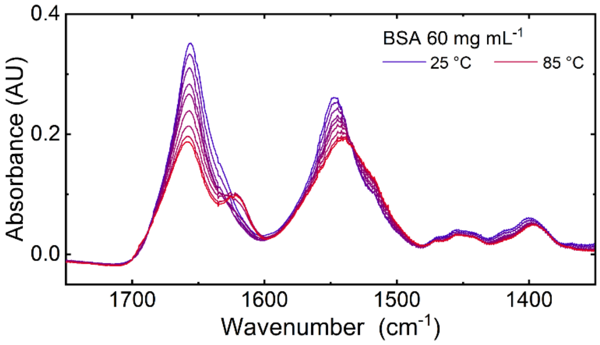

Fig. S1 Unsmoothed IR spectra of 60 mg mL^{-1} BSA between 25-85 °C, showing the full range of wavenumbers covered by the ChemDetect (1350 cm^{-1} -1450 cm^{-1}). This range covers both, the Amide I and Amide II bands, which are of interest for proteins

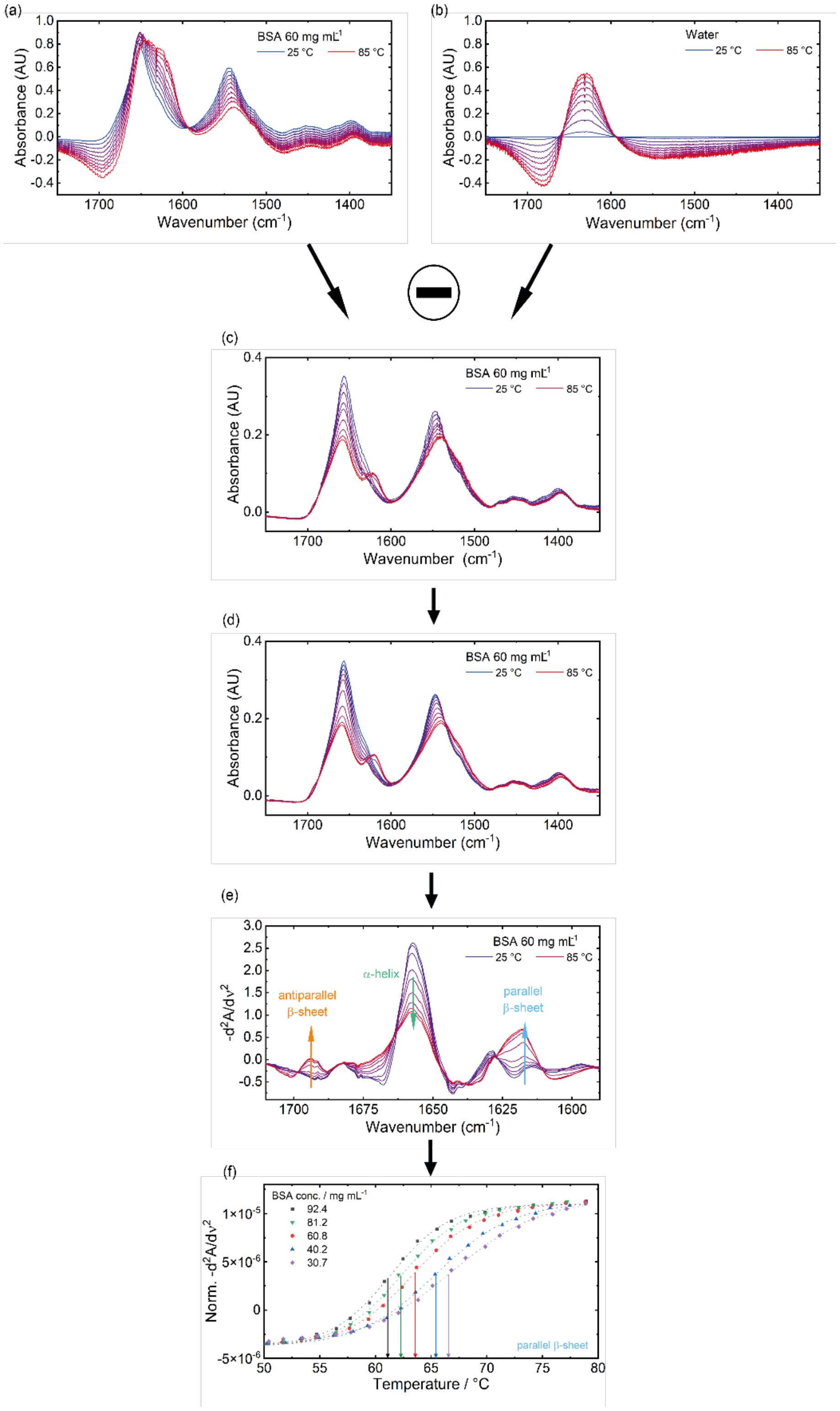

Fig. S2 Unsmoothed IR spectra of (**a**) 60 mg mL^{-1} BSA and (**b**) water spectra between 25-85 °C. Both, water and protein show changing spectral signatures at increasing temperatures. Therefore, in the next step, water spectra at corresponding temperatures are subtracted from the protein spectra to obtain (**c**) IR spectra depicting the change of proteins. Subsequently the spectra are (**d**) smoothed and (**e**) the second derivative is calculated. (**f**) Second derivative band heights at 1617, 1655 and 1692 cm^{-1} are plotted against temperature and a sigmoidal fit is applied to the obtained progressions. Denaturation temperature is determined as the inflection point of the sigmoidal shapes

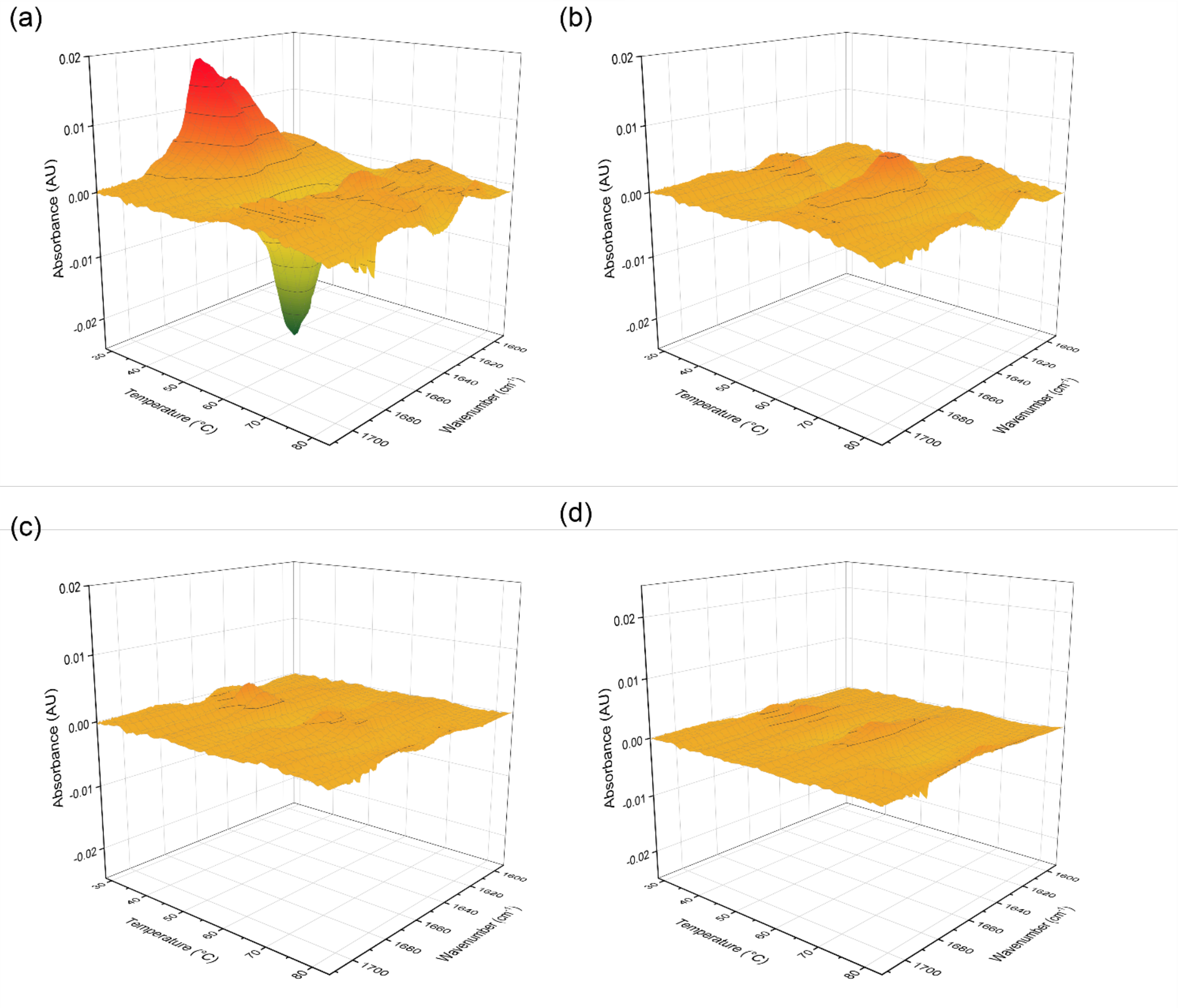

Fig. S3 3D error matrices containing residuals after performing MCR-ALS considering (**a**) 2 (**b**) 3 (**c**) 4 components and (**d**) 5 components during the denaturation of BSA

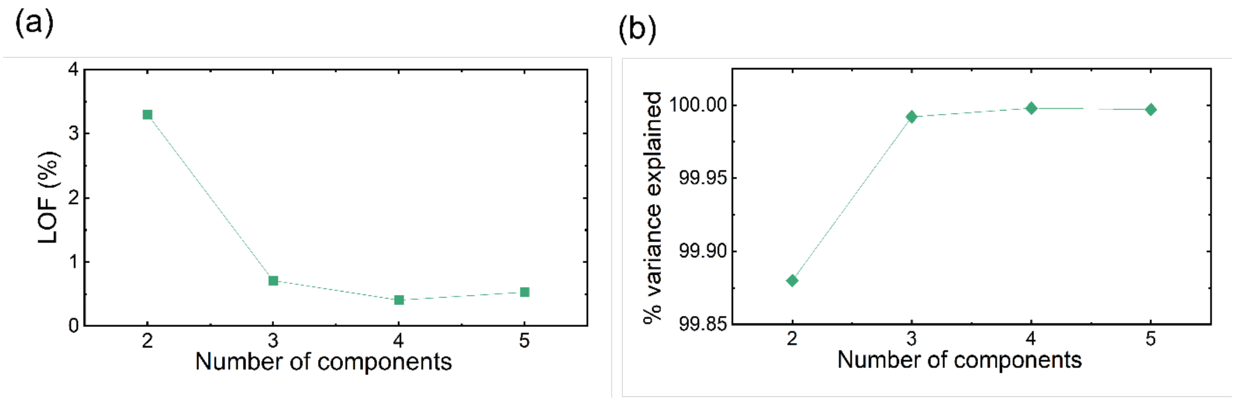

Fig. S4 Progression of LOF and percentage variance explained with different number of components for MCR-ALS

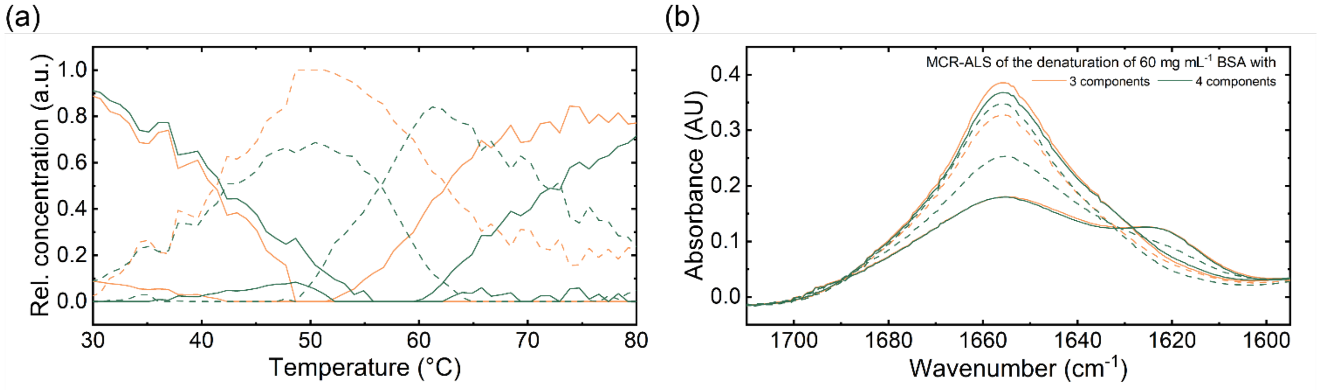

Fig. S5 (**a**) Concentration profiles of the secondary structure components versus temperature, resolved through MCR-ALS while choosing 3 (orange) and 4 (green) components respectively (**b**) Spectral profiles of the components of thermal denaturation of BSA obtained by choosing 3 (orange) and 4 (green) components

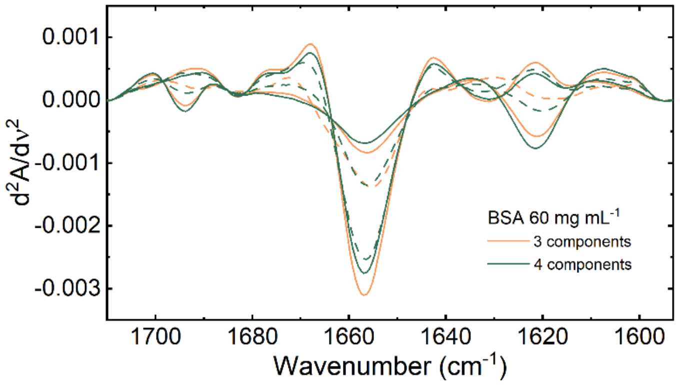

Fig. S6 Second derivative spectra of the three (orange) and four (green) component systems of the thermal denaturation of BSA modelled by MCR-ALS. The dashed lines in both cases represent the spectra of intermediate structures

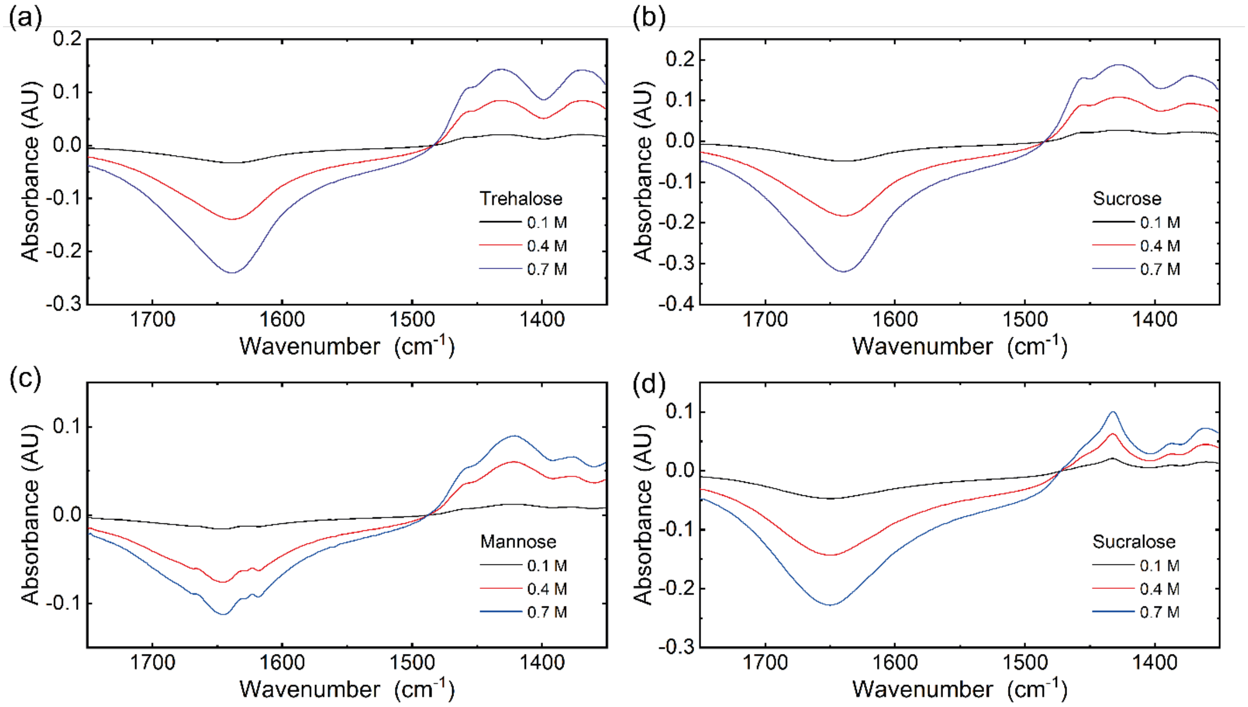

Fig. S7 Concentration dependent spectra of different sugars with respect to water

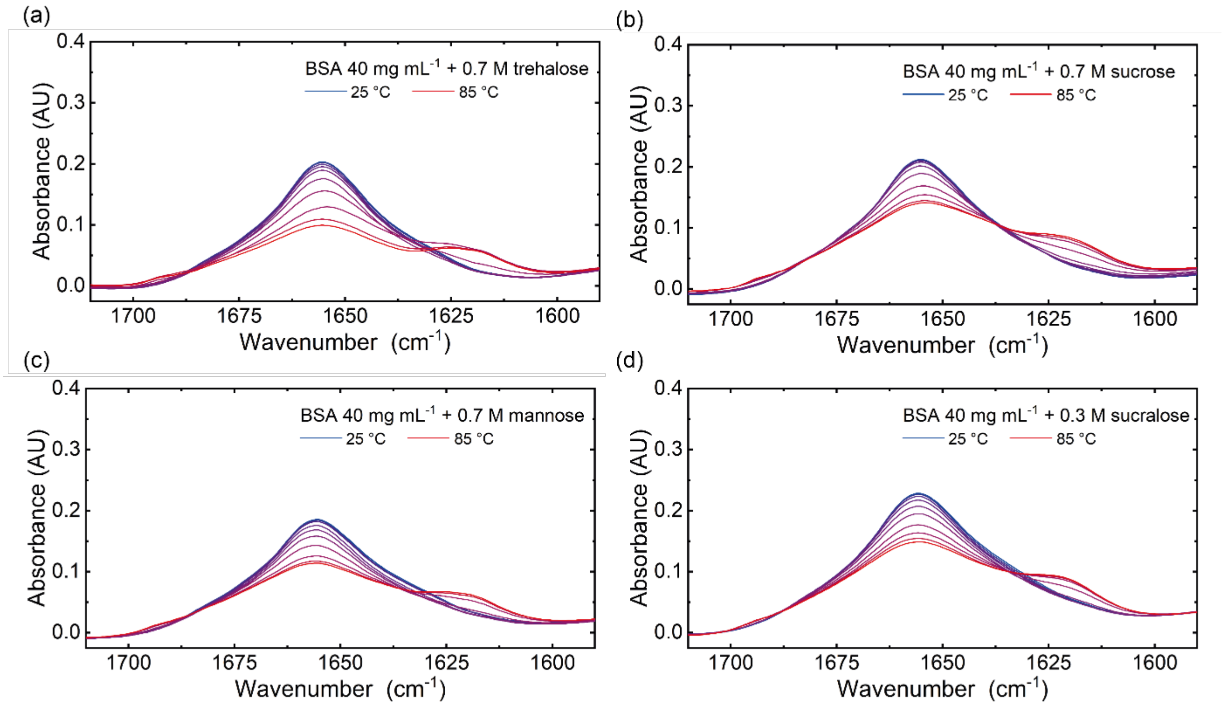

Fig. S8 Temperature dependent IR spectra of BSA in the presence of four different sugars showing that the band position is not affected by the presence of a stabilizing agent
